# Limited genetic diversity in *Salmonella enterica *Serovar Enteritidis PT13

**DOI:** 10.1186/1471-2180-7-87

**Published:** 2007-10-01

**Authors:** Adam B Olson, Ashleigh K Andrysiak, Dobryan M Tracz, Jean Guard-Bouldin, Walter Demczuk, Lai-King Ng, Anne Maki, Frances Jamieson, Matthew W Gilmour

**Affiliations:** 1 National Microbiology Laboratory, Public Health Agency of Canada, Winnipeg, MB, Canada; 2 United States Department of Agricultural, Agricultural Research Service, Athens, GA, USA; 3 Ontario Central Public Health Laboratory, Ministry of Health and Long-Term Care, Toronto, ON, Canada; 4 Department of Medical Microbiology, University of Manitoba, Winnipeg, MB, Canada

## Abstract

**Background:**

*Salmonella enterica *serovar Enteritidis has emerged as a significant foodborne pathogen throughout the world and is commonly characterized by phage typing. In Canada phage types (PT) 4, 8 and 13 predominate and in 2005 a large foodborne PT13 outbreak occurred in the province of Ontario. The ability to link strains during this outbreak was difficult due to the apparent clonality of PT13 isolates in Canada, as there was a single dominant pulsed-field gel electrophoresis (PFGE) profile amongst epidemiologically linked human and food isolates as well as concurrent sporadic strains. The aim of this study was to perform comparative genomic hybridization (CGH), DNA sequence-based typing (SBT) genomic analyses, plasmid analyses, and automated repetitive sequence-based PCR (rep-PCR) to identify epidemiologically significant traits capable of subtyping *S*. Enteritidis PT13.

**Results:**

CGH using an oligonucleotide array based upon chromosomal coding sequences of *S. enterica *serovar Typhimurium strain LT2 and the *Salmonella *genomic island 1 successfully determined major genetic differences between *S*. Typhimurium and *S*. Enteritidis PT13, but no significant strain-to-strain differences were observed between *S*. Enteritidis PT13 isolates. Individual loci (*safA *and *fliC*) that were identified as potentially divergent in the CGH data set were sequenced in a panel of *S*. Enteritidis strains, and no differences were detected between the PT13 strains. Additional sequence-based typing was performed at the *fimA*, *mdh*, *manB*, *cyaA*, *citT*, *caiC*, *dmsA*, *ratA *and STM0660 loci. Similarly, no diversity was observed amongst PT13 strains. Variation in plasmid content between PT13 strains was observed, but macrorestriction with B*gl*II did not identify further differences. Automated rep-PCR patterns were variable between serovars, but *S*. Enteritidis PT13 strains could not be differentiated.

**Conclusion:**

None of the methods identified any significant variation between PT13 strains. Greater than 11,300 base pairs of sequence for each of seven *S*. Enteritidis PT13 strains were analyzed without detecting a single polymorphic site, although diversity between different phage types of *S*. Enteritidis was observed. These data suggest that Canadian *S*. Enteritidis PT13 strains are highly related genetically.

## Background

*Salmonella enterica *serovar Enteritidis (*S*. Enteritidis) is a foodborne pathogen transmitted to humans predominately through contaminated eggs and other poultry food products [1]. *S*. Enteritidis was an infrequently reported serovar until the mid- to late 1980's when it emerged as a common cause of salmonellosis in European countries and then worldwide [2-4]. By the 1990's *S*. Enteritidis replaced *S. enterica *serovar Typhimurium (S. Typhimurium) as the most common serovar isolated from humans in many countries [5,3,4,7]. In Canada, *S*. Enteritidis has been among the top three reported *Salmonella *serovars resulting in human disease since 1998, accounting for between 12–21% of infections caused by *Salmonella *[8]. *S*. Enteritidis is subtyped by a phage typing scheme implemented by Ward *et al*., [9], and this method has identified regionally endemic *S*. Enteritidis subtypes as well fluctuations in the predominate subtypes. For example, *S*. Enteritidis phage type 13 (PT13) was the eighth most common phage type identified in Canada from 1982–1992, but by 1998 PT13 was ranked the third most common and has subsequently remained one of the top five most prevalent phage types [8].

Phage typing of *S*. Enteritidis utilizes a set of sixteen bacteriophage to generate a lytic pattern to group strains [9]. Typing methods based upon comparisons of whole genomic DNA, plasmid DNA or specific genetic determinants have also been used in place of phage typing or as supplementary techniques. The methods most frequently used to subtype *S*. Enteritidis include pulsed-field gel electrophoresis (PFGE), plasmid profiling and restriction-hybridization based ribotyping, which have each been applied with varying degrees of success [10-16]. A single PFGE macrorestriction profile often predominated in strains of the same phage type or even amongst multiple phage types [10,13,17-19]. Alternatively, a group of strains of the same phage type can have multiple restriction patterns [20,21]. Ribotyping based upon restriction analysis of the rRNA operon has utilized several schemes, such as *Pvu*II or a double digestion with *Pst*I and *Sph*I [22]. The latter combination has been used to subtype strains of the same phage type, however, this discrimination was not always epidemiologically significant [17]. Plasmid profiles have indicated divergence between strains represented by the presence or absence of various low molecular weight plasmids and the 55 to 60 kbp *S*. Enteritidis virulence plasmid [11,12,23], which is similar to the 94 kbp pSLT virulence plasmid of *S*. Typhimurium [24]. Combinations of these methods have been successfully used to subtype outbreak from non-outbreak strains of *S*. Enteritidis [25,11,13,15,18].

DNA microarray-based comparative genomic hybridization (CGH) and sequence-based typing (SBT) have recently been used to determine genetic relatedness between *S*. Enteritidis strains, and there was generally a lack of diversity for strains of the same phage type [26,24-29]. SBT of 24 clinical and poultry-related *S*. Enteritidis strains in a scheme comprised of segments of the 16S rRNA, *manB*, *glnA *and *pduF *loci detected 2, 37, 3 and 3 polymorphic sites, respectively [28]. Similarly, a SBT scheme evaluating *manB*, *mdh *and *fimA *encoded by 7 *S*. Enteritidis strains revealed 2, 2 and 0 polymorphic sites, respectively [26]. Typing of the *misL*, *spaM *and *spaN *loci encoding cell surface-associated proteins did not identify differences in four PT13 strains, but identified 2 polymorphic sites in PT4 [29]. CGH analyses of a diverse collection of 27 *S*. Enteritidis strains using a microarray composed of *S*. Typhimurium, *S*. Typhi, *S*. Paratyphi A and *S*. Enteritidis PT4 probes identified few genetic differences between these strains, including strains isolated before the emergence of *S*. Enteritidis as a major foodborne *Salmonella *[24]. The genetic differences were predominantly at phage-encoding loci (ST64B and Fels-2) but no significant delineation amongst *S*. Enteritidis strains of the same phage type was observed [24]. CGH analyses of PT13a and PT4 strains similarly identified no significant strain-to-strain variation, and these phage types were also distinguished by ST64B and Fels bacteriophage genetic content [27].

*S*. Enteritidis PT13 was identified in a large foodborne outbreak in the Canadian province of Ontario in 2005. Over 700 cases of gastroenteritis were reported between October and December 2005 and these were associated with the consumption of contaminated mung bean sprouts. A single PFGE profile (SENXAI.0038; SENBNI.0016) predominated amongst the outbreak-associated strains, and this profile was also seen in concurrent and preceding sporadic human-clinical and poultry-related isolates. With the apparent clonality of PT13 isolates, it was difficult to support epidemiological links during the outbreak using PFGE-based typing. The aim of this study was to perform CGH and SBT genomic analyses in parallel with other typing methodologies to identify epidemiologically significant markers for subtyping *S*. Enteritidis PT13.

## Results

**Figure 1:**
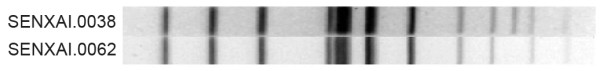
Pulsed-field gel electrophoresis of *S*. Enteritidis PT13 using *Xba*I. Two macrorestriction patterns (SENXAI) were observed, and are presented with high to low molecular weight fragments from left to right.

In response to the large outbreak of *S*. Enteritidis PT13, all *S*. Enteritidis PT13 strains with PFGE data reported to the National Microbiology Laboratory were retrospectively analyzed. This set of 32 strains comprised sporadic human-clinical isolates and also poultry-related agri-food isolates submitted by provincial public health laboratories in Alberta, British Columbia, Ontario and Québec. Two PFGE patterns were observed: 30 strains had the *Xba*I macrorestriction profile SENXAI.0038 and 2 strains produced the related pattern SENXAI.0062 (Fig. 1). PFGE with *Bln*I was performed for 19 of these strains and all were pattern SENBNI.0016. The inability to differentiate strains by PFGE necessitated the need to attempt additional subtyping methodologies for the differentiation of both outbreak and sporadic *S*. Enteritidis PT13 strains.

### Comparative genomic hybridization

**Figure 2:**
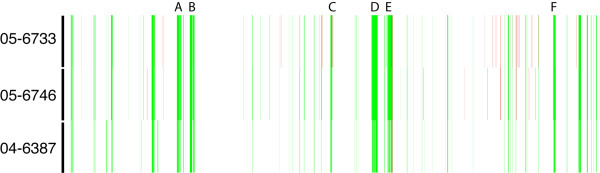
DNA microarray-based comparative genomics of *S*. Enteritidis PT13. Array probes represent the linear order of *S*. Typhimurium LT2 coding sequences from left to right, with the custom Salmonella genomic island 1 (SGI1) at the far-left side. White denotes similarity to LT2, green denotes putative divergence and red represents putative duplication or copy number change. Clusters of bacteriophage-related determinants that are divergent in *S*. Enteritidis compared to *S*. Typhimurium: A, STM893–929 (Fels-1 prophage); B, STM1005–1024 (Gifsy-2 prophage); C, STM2230–2240 (putative phage); D, STM2589–2636 (Gifsy-1 prophage); E, STM2732–2772 (Fels-2 prophage); F, STM4198–4218 (putative phage).

Microarray-based CGH was performed on *S*. Enteritidis strains to identify genetic differences between strains that would potentially serve as molecular markers for subtyping *S*. Enteritidis PT13 strains. An oligonucleotide array representing all coding sequences from the sequenced genome of *S*. Typhimurium LT2 and all coding sequences from SGI1 [30] was used in two-colour CGH experiments with *S*. Typhimurium LT2 genomic DNA as a reference and *S*. Enteritidis genomic DNA as the test strain. Seven PT13 strains were analyzed, including: human isolate 05-6733 associated with the foodborne outbreak; strain 05-6746 isolated from mung bean sprouts presumptively identified as the causative food agent; and five other sporadic PT13 strains isolated from human and poultry sources that were recovered concurrently with the outbreak or previously in other regions of Canada (see Table 1). The CGH analyses of PT13 strains individually indicated the genetic content of the strains in comparison to *S*. Typhimurium LT2, and the majority of the differences corresponded to genes encoded by bacteriophages Fels-1, Fels-2, Gifsy-1 and Gifsy-2, and also putative phage-related coding sequences STM2230–2240 and STM4198–4217 (Fig. 2). The oligonucleotides probes for SGI1 indicated that this determinant was absent in all strains, and accordingly, all examined *S*. Enteritidis strains were sensitive to antibiotics. The relative genetic content of *S*. Enteritidis strains identified by microarray analysis could also be used to compare between PT13 strains, and few coding sequences were putatively divergent between PT13 strains. DNA sequencing was performed at *fliC *and *safA *(both of which were putatively divergent in the CGH dataset), and each locus was identical for all strains PT13 strains (Table 1). These data suggested that differences in genetic content between PT13 strains in the CGH dataset were partly due to technical variation rather than true biological variation.

**Table 1:** Sequence-based typing of *Salmonella enterica *serovar Enteritidis. The number of polymorphic sites, in reference to PT13 isolate 05-6733, are indicated for each examined locus. The number of base pairs analyzed for each locus is indicated in brackets. Strains were isolated in 2004, 2005 or 2006, and these dates correspond to the first two digits in the strain identification number. BC, British Columbia; NS, Nova Scotia; ON, Ontario; QC, Québec

Strain	Source	PT	*Bgl*II PP^a^	*Xba*I PFGE	*fliC *(448)	*safA *(882)	*manB *(713)	*fimA *(720)	*mdh *(809)	*caiC *(834)	*dmsA *(837)	*citT *(827)	*ratA *(837)	STM0660 (1091)	*cyaA *(3392)
04-6191	Chicken, QC	13	1	0038	0	0	0	0	0	0	0	0	0	0	0
04-6387	Chicken, QC	13	1	0038	0	0	0	0	0	0	0	0	0	0	0
04-7505	Human, BC	13	1	0038	0	0	0	0	0	0	0	0	0	0	0
05-6746	Mung Bean, ON	13	1	0038	0	0	0	0	0	0	0	0	0	0	0
05-0513	Human, BC	13	1	0038	0	0	0	0	0	0	0	0	0	0	0
05-1219	Chicken, QC	13	0	0062	0	0	0	0	0	0	0	0	0	0	0
05-6733	Human, ON	13	1	0038	0	0	0	0	0	0	0	0	0	0	0
06-1230	Human, NS	1	1	0001	0	0	1	0	0	2	0	1	1	1	1
06-1751	Human, NS	1	2	0001	0	0	1	0	0	2	0	1	1	1	1
06-1226	Human, NS	4	1	0001	0	0	1	0	0	2	0	1	1	1	1
06-1231	Human, NS	4	0	0002	0	0	1	0	0	2	0	1	1	1	1
Tm LT2	ATCC 700720		3		222^b^	128^b^	6	5	7	25	5	4	3	29	10 (2547)

a. Plasmid profile as determined by digestion of plasmid DNA with restriction enzyme *Bgl*II; 0 = strains did not possess large plasmid and had no DNA fragments when digested with *Bgl*II

b. Individual polymorphisms are in addition to insertions and deletions, in reference to PT13 isolate 05-6733.

### Sequence-based typing

Due to the lack of observable differences in genetic content between PT13 using CGH, additional molecular methods were attempted to differentiate these strains. A SBT scheme targeting *manB*, *mdh *and *fimA *[26] did not identify sequence type differences amongst *S*. Enteritidis PT13 (Table 1). A single nucleotide polymorphism (SNP) was observed at the *manB *locus for both *S*. Enteritidis PT1 and PT4 compared to *S*. Enteritidis PT13. Previously, multiple SNPs have been observed between *S*. Enteritidis strains at the *cyaA *locus [31] and additional comparative analyses of *S*. Enteritidis phenotypic subpopulations that were descended from a common parent by whole genome mutational mapping identified polymorphisms, including small deletions, non-synonymous amino acid changes, and altered terminal codons [32]. From this data set, we sequenced for each strain in our *S*. Enteritidis panel the *ratA*, *citT*, *dmsA*, *caiC *and STM0660 loci encoding membrane proteins or metabolic cellular functions, and similarly no polymorphisms were observed between PT13 strains. A total of 11,390 bp, including the *fliC *and *safA *loci examined after CGH analyses, was sequenced for each strain without observing any genetic differences between the *S*. Enteritidis PT13 strains. A total of 7 SNPs were found between *S*. Enteritidis PT1 and PT4 compared to *S*. Enteritidis PT13, and a multitude of polymorphic sites between *S*. Typhimurium LT2 and *S*. Enteritidis PT13 were observed (Table 1).

### Plasmid content and plasmid RFLP

**Figure 3:**
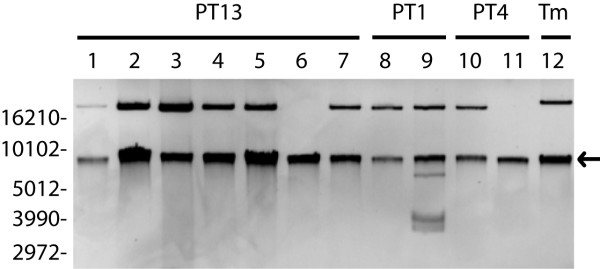
Plasmid profiles for *S*. Enteritidis strains used in this study. Preparations were not digested with restriction endonuclease. Lanes 1–7: *S*. Enteritidis PT13 strains 04-6191, 04-6387, 04-7505, 05-6746, 05-0513, 05-1219 and 05-6733 respectively. Lanes 8 and 9: *S*. Enteritidis PT1 strains 06-1230 and 06-1751. Lanes 10 and 11: *S*. Enteritidis PT4 strains 06-1216 and 06-1231. Lane 12: plasmid extracted from *S*. Typhimurium LT2. Supercoiled DNA ladder molecular weights are to the left of lane 1. Arrow indicates a chromosomal DNA fragment.

The population and characteristics of plasmids harbored by *Salmonella *has been used as a means to subtype these organisms [11]. Plasmid content was determined for the *S*. Enteritidis strains, as well as *S*. Typhimurium strain LT2, and in all plasmid preparations a chromosomal DNA fragment was observed (Fig. 3). A high molecular weight (HWM) plasmid was observed for all strains except PT13 strain 05-1219 and PT4 strain 06-1231. Accordingly, *S*. Typhimurium strain LT2 was known to harbor the 94 kbp virulence plasmid pSLT [24] and *S*. Enteritidis commonly harbors a ~60 kbp virulence plasmid [11]. Strains 05-1219 and 06-1231 that lacked the HMW virulence plasmid had a different PFGE pattern than the other *S*. Enteritidis PT13 and PT4 strains, respectively (Table 1 and Fig. 1). The only strain from which low molecular weight (LMW) plasmids were isolated was PT1 strain 06-1751. To confirm the presence of the *Salmonella *virulence plasmid in *S*. Enteritidis PT13, PCR screening for the *spv*C virulence determinant was performed. All strains encoded *spv*C (data not shown) except for the two *S*. Enteritidis strains that did not posses the HMW virulence plasmid.

**Figure 4:**
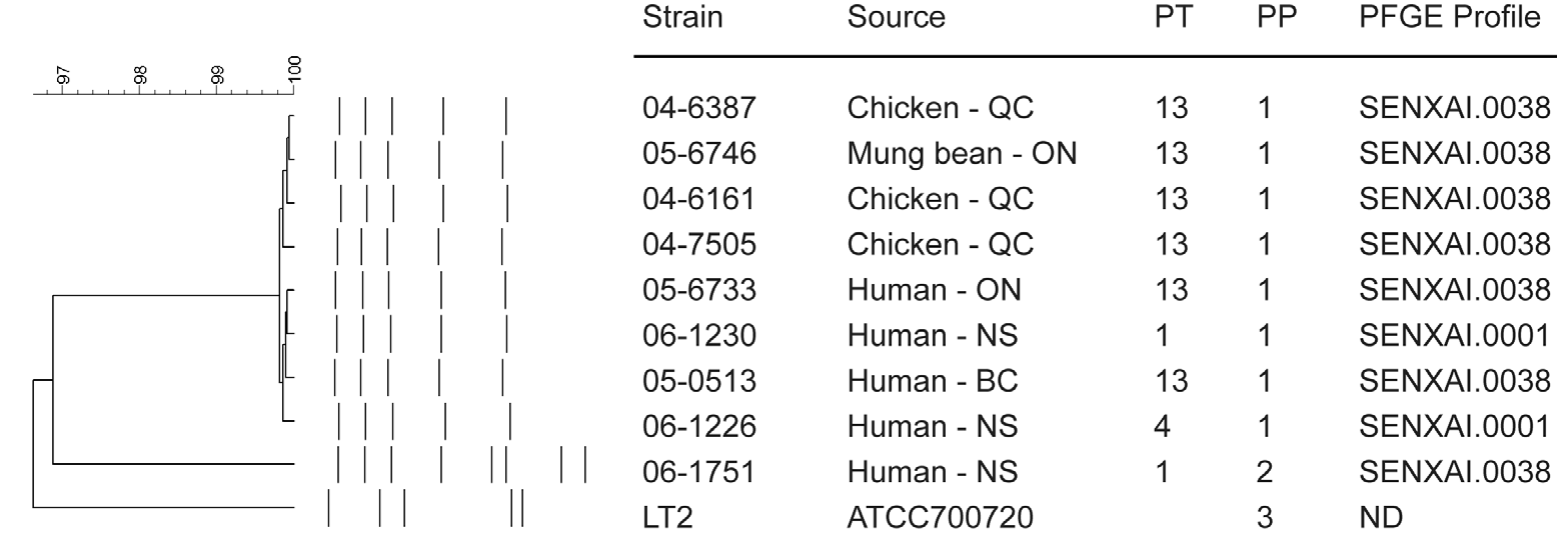
Plasmid RFLP patterns for *S*. Enteritidis PT13, PT1 and PT4 strains. Restriction fragment patterns generated with *Bgl*II were analyzed in BioNumerics version 4 and a dendrogram was created using the UPGMA method with a coefficient of correlation, 2% optimization and 12% position tolerance. PP; RFLP plasmid pattern, PT; phage type.

Restriction enzyme digestion of the plasmid preparations with *Bgl*II revealed that *S*. Enteritidis strains harboring the HMW plasmid had the same restriction fragment length polymorphism (RFLP) pattern (Fig. 4). The *S*. Enteritidis PT1 strain with LMW plasmids had a different plasmid pattern due to additional DNA fragments (corresponding to the LMW plasmids) and the fragments in the pattern contributed by the HMW plasmid were otherwise identical to those of strains that contained only the HMW plasmid. Additionally, the RFLP pattern produced by the S. Typhimurium pSLT differed from the *S*. Enteritidis HMW plasmid.

### Rep-PCR

**Figure 5:**
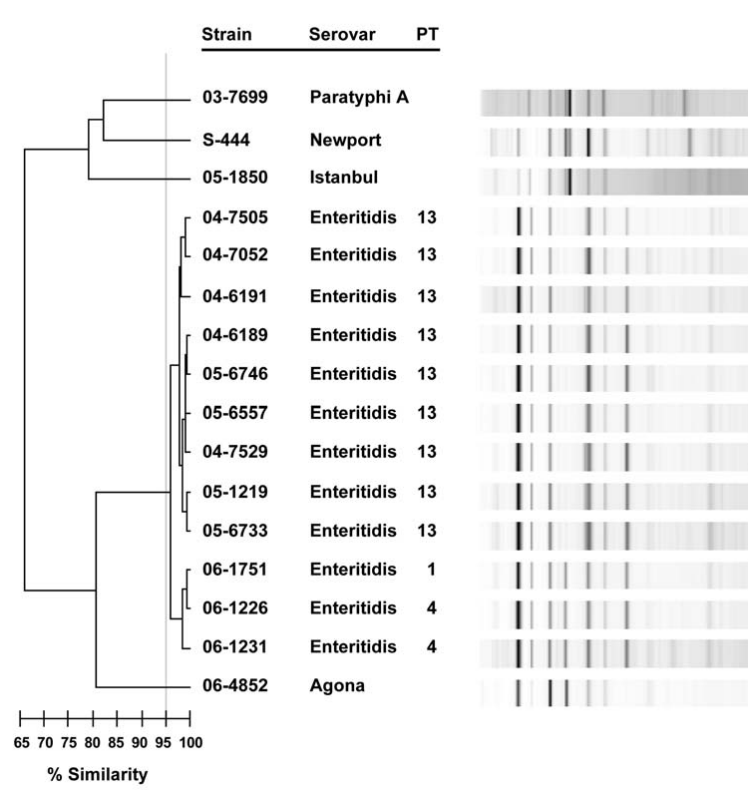
Comparison of *S*. Enteritidis and select *Salmonella *serovars by automated rep-PCR. The dendrogram represents the relatedness of strains based upon analyses of the amplification products using DiversiLab software. The vertical grey threshold line represents 95% similarity; PT = phage type.

Repetitive sequence-based PCR (rep-PCR) methods targeting non-coding repetitive sequences are useful for bacterial subtyping and this platform has been automated and reproducibility improved through commercially-available reagents in conjunction with a microfluidics station [33]. Automated rep-PCR was performed on *S*. Enteritidis PT13, PT1 and PT4 strains, and a small selection of other serovars was also included to illustrate the scope of differentiation at the serovar level. The rep-PCR amplicon patterns for all *S*. Enteritidis PT13 strains clustered together and were >98% related with no significant differences (Fig. 5). The *S*. Enteritidis PT1 and PT4 strains produced similar rep-PCR patterns as the PT13 strains (>95% relatedness) with a single amplicon being the predominant difference. The other examined serovars were between 78–81% related to the *S*. Enteritidis strains (Fig. 5).

## Discussion

Genetic homogeneity has been repeatedly observed for *S*. Enteritidis, particularly between strains of the same phage type [26,24,27]. Similarly, subtyping beyond phage type utilizing methods such as ribotyping, PFGE and plasmid profiling has generally only confirmed the clonal lineages discerned by phage typing without providing any further discriminatory power [10,34,29]. This lack of subtyping can prevent the establishment of absolute links to contaminated agri-food sources during outbreak trace-back investigations. During an outbreak of *S*. Enteritidis PT13 in Canada, the outbreak strain could not be distinguished from concurrent and geographically and temporally distinct isolates strains of *S*. Enteritidis PT13. CGH, SBT, plasmid profiling and rep-PCR were performed in an attempt to identify genetic markers suitable for subtyping *S*. Enteritidis PT13.

Clonally related phenotypic subpopulations of *S*. Enteritidis have been observed and discrete allelic variation at ribosomal and metabolic loci corresponded to these pathotypes [31,32]. In our CGH experiments, large clusters of genetic differences such as the absence of phage regions were discernible between *S*. Enteritidis and the reference *S*. Typhimurium for which the array was designed. However, more subtle genetic differences such as allelic variation between PT13 strains could not be confirmed, which was a finding similar to previous results [24,27]. This may have resulted from a lack of *S*. Enteritidis specific probes. Alternatively, identifying a reliable phenotypic or pathotype difference between PT13 strains could aid in the identification of subtle but definitive genetic differences that impact biology within a phage type.

Morales and colleagues hypothesized that genetic variation between *S*. Enteritidis strains is better studied by performing DNA sequencing for SNPs than through microarray-based comparative genomics [27]. Through sequencing, it is possible to produce *de novo *data for each strain instead of attempting to discern genomic content in the context of comparing hybridization data between two different strains, or possibly different serovars, as in this study (*S*. Typhimurium reference DNA versus *S*. Enteritidis test DNA). Although DNA sequencing provided a higher level of detail than CGH, sequencing of the loci that were previously observed to be divergent between *S*. Enteritidis pathotypes and other loci commonly used for sequence-based typing (total 11,390 bp per strain) did not identify any genomic differences between PT13 strains. These results are similar to other published results in that discrimination between serovars was possible, but variation within a phage type was not observed [29,26,35]. Alternatively, some loci were significantly variable between *S*. Enteritidis and *S*. Typhimurium (LT2), including *safA *(128 SNP), STM0660 (29 SNP) and *caiC *(25 SNP). These variable loci may prove to be useful as targets for molecular typing between *Salmonella *serovars. Our panel of strains was selected to include the outbreak-associated strains, concurrent sporadic clinical and agri-food strains, and for comparison to unrelated strains, isolates that were previously recovered in different Canadian provinces. If the incidence of SNPs is less than 1 in 10,000 bp it may be necessary to pursue a whole genome approach for detection of allelic variation because any one section of the genome or group of genes could be similar between clonally related PT13 strains relevant to human public health.

Plasmid profile analyses identified *S*. Enteritidis strains that lacked the HMW virulence plasmid or those that contained LMW plasmids, but these did not correspond to the outbreak-associated strains. Carriage of the HMW virulence plasmid were confirmed by testing for the presence of the *spvC *gene, which was previously observed on all *S*. Enteritidis plasmids of ~60 kbp [16]. The variation in plasmid content did influence the observed *Xba*I PFGE patterns, which were otherwise identical. Additionally, RFLP analyses of the plasmid preparations did not identify any further levels of genetic diversity within the plasmids, but did support that both *S*. Enteritidis PT1 and PT4 carry a similar HMW virulence plasmid as *S*. Enteritidis PT13. Furthermore, the usefulness of determining plasmid carriage as a means of subtyping was limited since the overall incidence of the HMW virulence plasmids in Canada was unknown and variation of this trait may be influenced by laboratory conditions (i.e. plasmid loss during culturing).

Rep-PCR has the potential to represent genetic differences contributed throughout the genome, unlike directed SBT and plasmid profiling, and targets different genetic features than PFGE. The *S*. Enteritidis PT13 strains examined in this study were not differentiated with this method and were greater than 97% related. Although there was some discrimination between the *S*. Enteritidis PT1 and PT4 strains and the *S*. Enteritidis PT13 strains (by a single band difference), there was still greater than 95% relatedness amongst these strains. Alternatively, a select number of other serovars were examined using automated rep-PCR and this method could differentiate between serovars, which is similar to a previous rep-PCR study [36].

## Conclusion

*S*. Enteritidis PT13 isolates in Canada were observed to be essentially genetically homogeneous after microarray-based comparisons did not identify overt differences between strains and DNA sequencing of eleven loci and 11,390 bp did not identify any polymorphisms. Furthermore, PFGE and plasmid RFLP patterns were identical for the majority of strains, except for some changes in plasmid content that affected restriction patterns of strains not associated with a large foodborne outbreak. Rep-PCR could discriminate between serovars and phage types, but not between outbreak-related and sporadic PT13 isolates. The apparent clonality of PT13 strains has implications for the ability to subtype this pathogen during outbreaks.

## Methods

### Bacterial strains

*Salmonella enterica *serovar Enteritidis strains were isolated from human, food and animal sources by public health laboratories in Ontario, Québec, British Columbia and Nova Scotia during 2004–2006 and submitted to the Enteric Diseases Program at the National Microbiology Laboratory. *S. enterica *serovar Typhimurium LT2 (ATCC 700720) was included as a positive control for CGH and SBT because many of the utilized oligonucleotides were designed using the genomic sequence data from this strain (GenBank accession number NC_003197). Confirmation of phage type was completed with bacteriophage stocks prepared at the National Microbiology Laboratory. Antibiotic sensitivity testing was completed using the National Antimicrobial Resistance Monitoring System (NARMS) recommended panel and the Sensititre broth dilution method.

### Plasmid Isolation and Analysis

Plasmids were purified from overnight culture using a Qiagen plasmid Midi kit (Qiagen, Mississauga, ON) according to manufacturer's directions with the following modification: plasmids were precipitated using 7.5 M ammonium acetate (Sigma-Aldrich, St. Louis, MO) in combination with isopropanol. Purified plasmid DNA (25 μl) was digested overnight at 37°C with 20 units of *Bgl*II (New England Biolabs, Pickering, ON). Resulting plasmid fragments were separated by electrophoresis on 0.7% Tris-acetate-EDTA agarose gels at 70 V for 6 hours in 1 × Tris-acetate-EDTA buffer (Gibco BRL, Paisley, Scotland). A 1 Kb Plus DNA Ladder (Invitrogen) and TrackIt™ λ DNA/*Hind*III fragments (Invitrogen) were used as molecular size standards. Gels were stained with ethidium bromide (2 μg/ml) and digital images were obtained using a Bio-Rad Gel Doc XR (Bio-Rad).

### PCR and Sequencing

Template DNA was prepared by centrifuging 1 mL of log-phase cultures grown in brain heart infusion (BHI) broth, the pellet was resuspended in 1 mL of TE buffer (Sigma, 10 mM Tris-HCl, 1 mM EDTA, pH 8.0) and boiling for 15 minutes. Boiled cells were pelleted and the supernatant was removed and used as DNA template in PCR reactions.

Oligonucleotide primer sequences used for DNA amplification and/or sequencing are presented in Table 2. PCR was performed with Platinum Taq (Invitrogen), following the manufacturer's directions. The thermocycling parameters for *fimA, manB *and *mdh *included an initial denaturation at 94°C for 5 minutes, 35 cycles of denaturation at 94°C for 30 seconds, annealing at 50°C for 30 seconds and extension at 68°C for 45 seconds, with a final extension at 68°C for 5 minutes. The annealing temperature for *caiC, dmsA, citT, ratA*, STM0660, was 55°C for 40 seconds and an extension of 60 seconds. The annealing temperature for *safA *and *fliC *was 50°C for 30 seconds and an extension of 30 seconds. For amplification of *cyaA*, the annealing temperature was 55°C for 60 seconds with an extension of 120 seconds. PCR products were purified using the QIAquick PCR purification kit (Qiagen) and sequenced using the same primers that generated these amplicons, with the exception of *cyaA *that required ten additional oligonucleotides targeting internal segments to provide complete coverage (Table 2). Sequencing was performed with an ABI3730 capillary electrophoresis instrument (Applied Biosystems, Foster City, CA). Multiple sequence alignments were completed using ClustalW [37] and Boxshade [38], and these data were deposited in GenBank with accession numbers EF113924–EF113956.

**Table 2:** Oligonucleotides used in this study. For oligonucleotides used in PCR the amplicon size is listed, and for those oligonucleotides used solely for DNA sequencing no product size is listed

Oligonucleotide	Target	Sequence (5' to 3')	Product size (bp)	Reference
sfimAF	*fimA*	TCAGGGGAGAAACAGAAAACTAAT	760	[25]
sfimAR		TCCCCGATAGCCTCTTCC		[25]
smanBF	*manB*	CATAACCCGATGGACTACAACG	893	[25]
smanBR		ACCAGCAGCCACGGGATCAT		[25]
smdhF	*mdh*	GATGAAAGTCGCAGTCCTCG	849	[25]
smdhR		TATCCAGCATAGCGTCCAGC		[25]
ccmBF	*ccmB*	TCACCCTGTTTCCGTTAAGC	430	This study
ccmBR		AAAATCAGCACCGGGACAC		This study
umuCF	*umuC*	TGACCACACTCGAGGAGATG	493	This study
umuCR		CAAACGATTTCCTGCTTTGC		This study
fljAF	*fljA*	GGCGAGAAGCTGAAATATGG	410	This study
fljAR		ATTTACGCCTGTCGTTTTGC		This study
safA2F	*safA*	TAAGAGGTGCTCTGATATATAG	959	This study
safA2R		ATAGGGTAATTCTGCGGGTTG		This study
caiC1F	*caiC*	GAATCGTTCGGCAGTTTAGC	874	This study
caiC1R		GTTTCAGTCATACCATAAGAGG		This study
citT1F	*citT*	GATGATTGTCGGTATGATCC	870	This study
citT1R		GTAATATCTTTCCACGGCAC		This study
dmsA1F	*dmsA*	ACTACGGTGATTACTCTTCC	869	This study
dmsA1R		CTGGTTAATCAAACAGTTGC		This study
ratA1F	*ratA*	GGCAAGATTCACAGCATTCAG	870	This study
ratA1R		TGGGCGGTATTTATCGTTCG		This study
STM0660F	STM0660	ACGATGTAGCCCATATTACG	1191	This study
STM0660R		CCTGGCGAAAGTATTCATCC		This study
spvC1	*spvC*	AACTCCTTGCACAACCAAATG	230	This Study
spvC2		ACCATATCCCTGAGCACACTG		This Study
Cya1sF	*cyaA*	CATTGACCATCCTAACATCCTTATAGAGAG	3331	[31]
Cya6sR		ACTGGCGATATCACTCAATAGCGG		[31]
Cya1sR	*cyaA*	ATGCTGCGTAGAACCACAGTCTTC		[31]
Cya2sF	*cyaA*	TGGATATCTGGGTGTGCCATCAGT		[31]
Cya2sR	*cyaA*	GACTTTACCCGGCAACGCTTCAAA		[31]
Cya3sF	*cyaA*	CGATTACCGGCGTTTACACCAT		[31]
Cya3sR	*cyaA*	ATATCTTTCGCCAGCAGACGTG		[31]
Cya4sF	*cyaA*	CGCAGGATATCGGCGTACTGA		[31]
Cya4sR	*cyaA*	GACGGCAATTTCACCTGGTGGTT		[31]
Cya5sF	*cyaA*	GCGTCGGGAAGTATTAAGCCAGTT		[31]
Cya5sR	*cyaA*	AGGTCGACAATACCGTTGCCCTTA		[31]
Cya6sF	*cyaA*	TACGTCTTCCAGCACCCGTCA		[31]

### Rep-PCR typing

Total bacterial genomic DNA was isolated from selected SE strains (Table 2) and a small selection of other *Salmonella *serovars (*S*. Agona strain 06-4852, *S*. Istanbul 05-1850, *S*. Newport S-444, and *S*. Paratyphi A 03-7699) using the Qiagen DNeasy extraction kit, following manufacturer's instructions. The DiversiLab *Salmonella *DNA fingerprinting kit (Bacterial Barcodes) was used for automated rep-PCR molecular typing using AmpliTaq DNA polymerase (Roche) and PCR conditions identified by Bacterial Barcodes. The rep-PCR amplicons were separated using an Agilent 2100 Bioanalyzer on the DiversiLab microfluidics DNA chip. DNA fingerprints were statistically analyzed using DiversiLab software using manufacturer's instructions.

### Comparative Genomic Hybridizations

DNA microarrays were constructed as previously described [39] from 4451 commercially-supplied oligonucleotides (Qiagen) representing coding sequences of *S*. Typhimurium LT2, with the custom addition of the putative open reading frames from SGI1. Each oligonucleotide was spotted in duplicate per slide. DNA was isolated from overnight cultures grown in BHI broth at 37°C using the alkaline lysis protocol [40]. Proteins were removed using phenol chloroform extractions in Phase lock gel tubes (Eppendorf, Westbury, NY) and purified DNA was fragmented in a Nebulizer (Invitrogen) as per manufacturer's protocols, with the exception that DNA was nebulized for 3 minutes at 10 psi to yield DNA fragments between 500 and 700 bp. Probe DNA was created by labeling the fragmented genomic DNA with Cy3- or Cy5-dCTP (Amersham, Baie d'Urfe, QC) by random priming using the Bioprime Labeling Kit (Invitrogen) following the manufacturer's directions. The concentration and labeling efficiencies were measured using the NanoDrop ND-1000 spectrophotometer (NanoDrop technologies, Wilmington, DE). For each array a Cy3 or Cy5-labeled reference probe DNA (always *S*. Typhimurium LT2) was combined with a Cy3 or Cy5-labeled test PT13 strain along with 20 μg of Salmon Sperm DNA and dried in a Vacufuge (Eppendorf). Each test-versus-reference comparison was performed in triplicate, with at least one of the slides hybridized as a dye swap. The DNA pellet was resuspended in 10 μl of ddH_2_0, denatured at 95°C for 5 minutes, and snap cooled on ice for 5 minutes prior to hybridization to the array.

Slides were prepared as previously published [39] with the exception that they were pre-hybridized at 42°C in DIG Easy-Hyb buffer (Roche Diagnostic, Laval, QC) for 45 minutes, followed by brief washes in dH_2_O and isopropanol. 50 μL of DIG Easy-Hyb buffer was added to each probe mixture and the entire volume was pipetted under an M-Series LifterSlip™ (Erie Scientific Co., Portsmouth, NH) onto the pre-hybridized slides. Slides were hybridized at 42°C in a hybridization chamber (Genetix, Hampshire, UK). Slides were sequentially washed as follows: Buffer 1 (1× SSC and 0.2% SDS: Amersham) for 6 minutes at 56°C; Buffer 2 (0.1× SSC and 0.2% SDS) for 4 minutes at room temperature; two washes for 2 minute with Buffer 3 (0.1× SSC). Slides were spun dry and scanned using the Agilent DNA microarray scanner (Agilent Technologies, Mississauga, ON).

Scanned slide images were analyzed as previously published [39] with the exception that following normalization and batch-effect removal, the data was antilog_2_-transformed to convert back to its original scale so that the log_2 _ratio between test and reference could be measured. Log ratios were averaged across all replicates per spot for each test versus reference comparison. GeneMaths XT software (Applied Maths, Austin, TX) was used for Hierarchical clustering of the data to identify overall genetic relatedness between examined strains, and identify specific loci that were putatively absent of divergent in individual *S*. Enteritidis strains.

## Abbreviations

Comparative genomic hybridization (CGH), High molecular weight (HMW), Low molecular weight (LMW), Phage type (PT), Pulsed field gel electrophoresis (PFGE), Restriction fragment length polymorphism (RFLP), RFLP Plasmid pattern (PP), *Salmonella enterica *serovar (*S*.), Sequence based typing (SBT), Single nucleotide polymorphism (SNP).

## Competing interests

The author(s) declares that there are no competing interests.

## Authors' contributions

ABO performed CGH, SBT and plasmid profile analyses for this study, assisted in the study design, and drafted the manuscript. AKA and DMT participated in plasmid isolations and analysis, rep-PCR analysis, and assisted in drafting and revising the manuscript. JGB provided access to whole genomic analysis of *S*. Enteritidis subpopulations and assisted in the sequence analysis. WD, AM, FJ and LKN assisted in conceiving the study, provided epidemiological data for strains and revised the manuscript. MWG supervised the project and drafted and revised the manuscript. All authors read and approved the final manuscript.

## References

[B1] St Louis ME, Morse DL, Potter ME, DeMelfi TM, Guzewich JJ, Tauxe RV, Blake PA (1988). The emergence of grade A eggs as a major source of Salmonella enteritidis infections. New implications for the control of salmonellosis. JAMA.

[B2] Poppe C, Saeed AM, Gast RK, Potter ME and Wall PG (1999). Epidemiology of Salmonella enterica Serovar Enteritidis. Salmonella enterica Serovar Enteritidis in Humans and Animals.

[B3] Munro DS, Girdwood RWA, Reilly WJ, Saeed AM, Gast RK, Potter ME and Wall PG (1999). Salmonella enterica Serovar Enteritidis in Scotland. Salmonella enterica Serovar Enteritidis in Humans and Animals.

[B4] Cogan TA, Humphrey TJ (2003). The rise and fall of Salmonella Enteritidis in the UK. J Appl Microbiol.

[B5] Tschape H, Liesegang A, Gericke B, Prager R, Rabsch W, Helmuth R, Saeed AM, Gast RK, Potter ME and Wall PG (1999). Ups and Downs of Salmonella enterica Serovar Enteritidis in Germany. Salmonella enterica Serovar Enteritidis in Humans and Animals.

[B6] Mishu B, Koehler J, Lee LA, Rodrigue D, Brenner FH, Blake P, Tauxe RV (1994). Outbreaks of Salmonella enteritidis infections in the United States, 1985-1991. J Infect Dis.

[B7] Angulo FJ, Swerdlow DL, Saeed AM, Gast RK, Potter ME and Wall PG (1999). Epidemiology of Human Salmonella enterica Serovar Enteritidis Infections in the United States. Salmonella enterica Serovar Enteritidis in Humans and Animals.

[B8] Demczuk W, Woodward D, Ahmed R, Clark C, Tabor H, Dore K, Ciampa N, Muckle A (2005). Laboratory surveillance data for enteric pathogens in Canada: Annual summary 2002 and 2003.

[B9] Ward LR, de Sa JD, Rowe B (1987). A phage-typing scheme for Salmonella Enteritidis. Epidemiol Infect.

[B10] Thong KL, Ngeow YF, Altwegg M, Navaratnam P, Pang T (1995). Molecular analysis of Salmonella Enteritidis by pulsed-field gel electrophoresis and ribotyping. J Clin Microbiol.

[B11] Pang JC, Chiu TH, Chiou CS, Schroeter A, Guerra B, Helmuth R, Tsen HY (2005). Pulsed-field gel electrophoresis, plasmid profiles and phage types for the human isolates of Salmonella enterica serovar Enteritidis obtained over 13 years in Taiwan. J Appl Microbiol.

[B12] Millemann Y, Lesage MC, Chaslus-Dancla E, Lafont JP (1995). Value of plasmid profiling, ribotyping, and detection of IS200 for tracing avian isolates of Salmonella Typhimurium and S. Enteritidis. J Clin Microbiol.

[B13] Liebana E, Garcia-Migura L, Breslin MF, Davies RH, Woodward MJ (2001). Diversity of strains of Salmonella enterica serotype Enteritidis from English poultry farms assessed by multiple genetic fingerprinting. J Clin Microbiol.

[B14] Liebana E, Garcia-Migura L, Clouting C, Clifton-Hadley FA, Breslin M, Davies RH (2003). Molecular fingerprinting evidence of the contribution of wildlife vectors in the maintenance of Salmonella Enteritidis infection in layer farms. J Appl Microbiol.

[B15] Liebana E, Clouting C, Garcia-Migura L, Clifton-Hadley FA, Lindsay E, Threlfall EJ, Davies RH (2004). Multiple genetic typing of Salmonella Enteritidis phage-types 4, 6, 7, 8 and 13a isolates from animals and humans in the UK. Vet Microbiol.

[B16] Soto SM, Rodriguez I, Rodicio MR, Vila J, Mendoza MC (2006). Detection of virulence determinants in clinical strains of Salmonella enterica serovar Enteritidis and mapping on macrorestriction profiles. J Med Microbiol.

[B17] Clark CG, Kruk TM, Bryden L, Hirvi Y, Ahmed R, Rodgers FG (2003). Subtyping of Salmonella enterica serotype Enteritidis strains by manual and automated PstI-SphI ribotyping. J Clin Microbiol.

[B18] Bakeri SA, Yasin RM, Koh YT, Puthucheary SD, Thong KL (2003). Genetic diversity of human isolates of Salmonella enterica serovar Enteritidis in Malaysia. J Appl Microbiol.

[B19] Ahmed R, Soule G, Demczuk WH, Clark C, Khakhria R, Ratnam S, Marshall S, Ng LK, Woodward DL, Johnson WM, Rodgers FG (2000). Epidemiologic typing of Salmonella enterica serotype Enteritidis in a Canada-wide outbreak of gastroenteritis due to contaminated cheese. J Clin Microbiol.

[B20] Ridley AM, Threlfall EJ, Rowe B (1998). Genotypic characterization of Salmonella enteritidis phage types by plasmid analysis, ribotyping, and pulsed-field gel electrophoresis. J Clin Microbiol.

[B21] Powell NG, Threlfall EJ, Chart H, Rowe B (1994). Subdivision of Salmonella enteritidis PT 4 by pulsed-field gel electrophoresis: potential for epidemiological surveillance. FEMS Microbiol Lett.

[B22] Landeras E, Gonzalez-Hevia MA, Alzugaray R, Mendoza MC (1996). Epidemiological differentiation of pathogenic strains of Salmonella Enteritidis by ribotyping. J Clin Microbiol.

[B23] Liebisch B, Schwarz S (1996). Molecular typing of Salmonella enterica subsp. enterica serovar Enteritidis isolates. J Med Microbiol.

[B24] Porwollik S, Santiviago CA, Cheng P, Florea L, McClelland M (2005). Differences in gene content between Salmonella enterica serovar Enteritidis isolates and comparison to closely related serovars Gallinarum and Dublin. J Bacteriol.

[B25] Tassios PT, Markogiannakis A, Vatopoulos AC, Katsanikou E, Velonakis EN, Kourea-Kremastinou J, Legakis NJ (1997). Molecular epidemiology of antibiotic resistance of Salmonella Enteritidis during a 7-year period in Greece. J Clin Microbiol.

[B26] Sukhnanand S, Alcaine S, Warnick LD, Su WL, Hof J, Craver MP, McDonough P, Boor KJ, Wiedmann M (2005). DNA sequence-based subtyping and evolutionary analysis of selected Salmonella enterica serotypes. J Clin Microbiol.

[B27] Morales CA, Porwollik S, Frye JG, Kinde H, McClelland M, Guard-Bouldin J (2005). Correlation of phenotype with the genotype of egg-contaminating Salmonella enterica serovar Enteritidis. Appl Environ Microbiol.

[B28] Kotetishvili M, Stine OC, Kreger A, Morris JG, Sulakvelidze A (2002). Multilocus sequence typing for characterization of clinical and environmental Salmonella strains. J Clin Microbiol.

[B29] Hudson CR, Garcia M, Gast RK, Maurer JJ (2001). Determination of close genetic relatedness of the major Salmonella enteritidis phage types by pulsed-field gel electrophoresis and DNA sequence analysis of several Salmonella virulence genes. Avian Dis.

[B30] Boyd D, Peters GA, Cloeckaert A, Boumedine KS, Chaslus-Dancla E, Imberechts H, Mulvey MR (2001). Complete nucleotide sequence of a 43-kilobase genomic island associated with the multidrug resistance region of Salmonella enterica serovar Typhimurium DT104 and its identification in phage type DT120 and serovar Agona. J Bacteriol.

[B31] Morales CA, Musgrove M, Humphrey TJ, Cates C, Gast R, Guard-Bouldin J (2007). Pathotyping of Salmonella enterica by analysis of single-nucleotide polymorphisms in cyaA and flanking 23S ribosomal sequences. Environ Microbiol.

[B32] (2007). Comparative analyses of S. Enteritidis phenotypic subpopulations. http://www.ncbi.nlm.nih.gov/genomes/static/Salmonella_SNPS.html.

[B33] Healy M, Huong J, Bittner T, Lising M, Frye S, Raza S, Schrock R, Manry J, Renwick A, Nieto R, Woods C, Versalovic J, Lupski JR (2005). Microbial DNA typing by automated repetitive-sequence-based PCR. J Clin Microbiol.

[B34] Olsen JE, Skov MN, Threlfall EJ, Brown DJ (1994). Clonal lines of Salmonella enterica serotype Enteritidis documented by IS200-, ribo-, pulsed-field gel electrophoresis and RFLP typing. J Med Microbiol.

[B35] Fakhr MK, Nolan LK, Logue CM (2005). Multilocus sequence typing lacks the discriminatory ability of pulsed-field gel electrophoresis for typing Salmonella enterica serovar Typhimurium. J Clin Microbiol.

[B36] Bennasar A, de Luna G, Cabrer B, Lalucat J (2000). Rapid identification of Salmonella typhimurium, S. enteritidis and S. virchow isolates by polymerase chain reaction based fingerprinting methods. Int Microbiol.

[B37] (2007). ClustalW alignment tool. http://www.ebi.ac.uk/clustalw/.

[B38] (2007). Boxshade. http://www.ch.embnet.org.

[B39] Golding GR, Olson AB, Doublet B, Cloeckaert A, Christianson S, Graham MR, Mulvey MR (2007). The effect of the Salmonella genomic island 1 on in vitro global gene expression in Salmonella enterica serovar Typhimurium LT2. Microbes Infect.

[B40] Sambrook J, Russell DW (2001). Molecular Cloning: A Laboratory Manual.

